# Parental depressive symptoms, children’s emotional and behavioural problems, and parents’ expressed emotion—Critical and positive comments

**DOI:** 10.1371/journal.pone.0183546

**Published:** 2017-10-18

**Authors:** Lamprini Psychogiou, Nicholas J. Moberly, Elizabeth Parry, Selina Nath, Angeliki Kallitsoglou, Ginny Russell

**Affiliations:** 1 School of Psychology, University of Exeter, Exeter, United Kingdom; 2 Institute of Psychiatry, Psychology & Neuroscience, Kings College London, London, United Kingdom; 3 School of Education, University of Roehampton, London, United Kingdom; 4 University of Exeter Medical School, University of Exeter, Exeter, United Kingdom; TNO, NETHERLANDS

## Abstract

This longitudinal study examined whether mothers’ and fathers’ depressive symptoms predict, independently and interactively, children’s emotional and behavioural problems. It also examined bi-directional associations between parents’ expressed emotion constituents (parents’ child-directed positive and critical comments) and children’s emotional and behavioural problems. At time 1, the sample consisted of 160 families in which 50 mothers and 40 fathers had depression according to the Structured Clinical Interview for DSM-IV. Children’s mean age at Time 1 was 3.9 years (*SD* = 0.8). Families (*n* = 106) were followed up approximately 16 months later (Time 2). Expressed emotion constituents were assessed using the Preschool Five Minute Speech Sample. In total, 144 mothers and 158 fathers at Time 1 and 93 mothers and 105 fathers at Time 2 provided speech samples. Fathers’ depressive symptoms were concurrently associated with more child emotional problems when mothers had higher levels of depressive symptoms. When controlling for important confounders (children’s gender, baseline problems, mothers’ depressive symptoms and parents’ education and age), fathers’ depressive symptoms independently predicted higher levels of emotional and behavioural problems in their children over time. There was limited evidence for a bi-directional relationship between fathers’ positive comments and change in children’s behavioural problems over time. Unexpectedly, there were no bi-directional associations between parents’ critical comments and children’s outcomes. We conclude that the study provides evidence to support a whole family approach to prevention and intervention strategies for children’s mental health and parental depression.

## Introduction

Emotional and behavioural problems tend to occur as early as the toddler and preschool years, show persistence across development, cause impairments in many areas of child functioning and predict a range of adverse outcomes [1–6]. Parents’ low socioeconomic status, fathers’ absence, children’s male gender and poor physical health have been identified as risk factors for child psychopathology [4, 6, 7– 8]. There is also considerable evidence showing that depression in mothers predicts increased emotional and behavioural problems in their children [9]. On average, fathers’ depressive symptoms rise significantly during the first five years of a child’s life [10] and, as with mothers, paternal depression puts their offspring at increased risk for emotional and behavioural problems [11]. There is a positive association between maternal and paternal depression [12] and therefore some children may be exposed to both parents’ depression, which may put children at higher risk compared to exposure to parental depression in one parent only [13]. This study examined whether mothers’ and fathers’ depressive symptoms independently and interactively predicted increased emotional and behavioural problems in preschool children. A secondary aim was to explore whether child’s gender would moderate the associations between parents’ depressive symptoms and children’s emotional and behavioural outcomes. While the current literature is inconsistent about the role of child gender, some evidence suggests that impact of parental depression might be stronger for children with the same gender as the depressed parent [9, 11].

Both genetic and environmental factors have been found to explain the link between parental depression and children’s emotional and behavioural problems [14, 15]. Among other environmental factors (e.g., increased stress, lack of social support), difficulties in parenting have been identified as a key mechanism of risk transmission. It is well established that depressed mothers interact with their children in a less positive way and depressed fathers display more negative and fewer positive parenting behaviours [16–19]. Empirical studies provide support for parent-driven effects and the influence of mothers’ and fathers’ parenting behaviour on child developmental outcomes. Denham *et al*. [20] found that parents’ use of proactive parenting was longitudinally associated with decreased levels of behavioural problems among their children. Ramchandani *et al*. [21] found that remote and disengaged father-infant interactions at 3 months predicted increased child behavioural problems at 1 year when controlling for both parents’ depression and other important characteristics. Studies also provide support for child-driven effects. Anderson, Lytton and Romney [22] found that, compared to control boys, boys with conduct disorder elicited more demands and negative responses when interacting with their own mothers, mothers of other children with conduct problems and mothers of control children. The transactional model [23] theorizes that there are “bidirectional links” between parent’s and child’s behaviours. In support, a number of longitudinal studies have found reciprocal associations between parenting and children’s behavioural problems [24–26].

Parental expressed emotion is an index of the emotional quality of the parent-child relationship and includes a critical and an emotional over-involvement dimension [27]. Parental expressed emotion-criticism is high toward children with psychiatric disorders. Cartwright et al. [28] compared parental expressed emotion toward children with ADHD and their non-ADHD siblings and found that expressed emotion was higher toward ADHD children. Importantly, expressed emotion-criticism has been found to predict worse developmental outcomes in clinical populations. For example, a recent longitudinal study found that children with ADHD did not show declines in symptomatology observed in population-based samples when their parents expressed high and persistent expressed emotion-criticism [29]. There is also evidence indicating that parental psychopathology might play a role in high expressed emotion. The critical dimension of expressed emotion has been found to be significantly higher in depressed mothers [30–31]. However, it remains unresolved whether there are bi-directional influences between parents’ expressed emotion and child’s behaviour.

Based on previous literature, we predicted that there would be both independent and interactive effects of maternal and paternal depressive symptoms in predicting child problems both concurrently and longitudinally and that these effects would remain significant when controlling parent age, education and child gender. We predicted that there would be a multiplier effect such that, when both parents are high in depressive symptoms, child outcomes would be worse than would be expected after considering the additive effects of each parents’ depressive symptoms. We also predicted that child gender would moderate the links between parents’ depressive symptoms and children’s emotional and behavioural problems. Based on previous studies [9,11], we predicted that mothers’ depressive symptoms would have stronger effects on girls’ emotional and behavioural problems while fathers’ depressive symptoms would have stronger effects on boys, and there would be bi-directional longitudinal associations between parents’ expressed emotion constituents (as measured by levels of their critical/positive comments about their children) and child emotional and behavioural problems when controlling for baseline measures of outcome and child gender, parent education, age and parents’ depressive symptoms. In particular, we hypothesised that parents’ critical comments at Time 1 would predict child behavioural problems at Time 2 while child behavioural problems at Time 1 would predict parents’ critical comments at Time 2. We also predicted that parental positive comments at Time 1 would predict lower levels of child behavioural problems at Time 2 and child behavioural problems at Time 1 would predict lower levels of parental positive comments at Time 2. We expected similar effects for child emotional problems. This study focuses on preschool and early school years because during this period children have to learn to regulate their behaviour, symptoms of psychopathology stabilize, and the parent-child pair may start to engage in angry exchanges [24].

## Materials and methods

### Participants and procedure

The sample consisted of families who took part in a longitudinal study called Fathers in Focus. Because fathers were the main focus of the study, we established the sample by recruiting eligible fathers via nurseries, community centres and GP practices. Fathers (n = 1277) received an information leaflet, the Patient Health Questionnaire [32], a screening questionnaire that measures current depressive symptoms on a continuous scale and an invitation to participate in the study. Fathers’ partners were also invited to participate but mother’s participation was not among the study’s inclusion criteria. In total, 544 fathers returned the Patient Health Questionnaire (PHQ-9) and of those 319 expressed interest to participate in the study. Of the 319, 160 participated in the study, 68 decided not to participate when the researcher spoke to them over the telephone, 52 were not contactable and 39 did not meet the study’s inclusion criteria. Participants were included in the study if they were biological parents of children aged 3–5 years of age, were able to read and speak English and gave their consent to be visited by a researcher at their home. Participants were excluded if their child had a medical and neurological condition, language and cognitive delays. The study was approved by relevant National Health Service Ethics Committees (REC reference number: 11/H0102/6) and the University of Exeter, Psychology department ethics committee. The research procedures of the study are also discussed elsewhere [33].

At the first assessment (Time 1), the sample consisted of 160 families (160 fathers and 146 mothers). At the start of a home assessment, a trained psychologist collected written consent forms from the parents and provided information about the study and research procedures. Each parent provided information about depressive episodes using the Structural Clinical Interview for DSM-IV [34] to assess diagnosis of Major Depressive Episode in the last month (current depression) or at any other time (a history of depression). Parents were seen again 16 (*M* = 16.2, SD = 3.7; Time 2) months after the first assessment (*n* = 106 families; 106 fathers and 98 mothers; 66% response rate). There were no significant differences in child and parent characteristics between parents who remained in the study and parents who dropped out. The study duration spans the transition from the preschool to school age period during which children increasingly have to show appropriate self-regulation skills while behavioural problems become more stable with some emerging differences in their prevalence between boys and girls [1, 24]. Similar intervals between assessments have been used in previous studies testing the effects of parental depression on child outcomes [35].

At the first assessment (Time 1), 40 (25%) fathers and 50 (34%) mothers met criteria for current or previous Major Depressive Episode. All 50 (100%) mothers had a history of depression while out of the 40 depressed fathers, 7 (17%) had current depression and 33 (83%) had a history of depression. Fathers’ and mothers’ mean age was 39 and 36 years respectively. Most couples (95%) were married or living together and the overwhelming majority of fathers’ (95%) and mothers’ (95%) nationality was White British. Most of the participants had an undergraduate (27% of fathers and 31% of mothers) or postgraduate degree (31% of fathers and 29% of mothers). The mean age of children was 3.9 years (SD = 0.8) and the number of boys and girls was roughly equal (47% boys).

### Measures

#### Parents’ depressive symptoms

We used the Patient Health Questionnaire [32] to measure mothers’ and fathers’ symptoms of depression in the past two weeks. Items are rated on a 4-point scale ranging from 0 = *Not at all* to 3 = *Nearly every day*. A score of 20 and above indicates severe depression. A diagnosis of depression based on PHQ-9 is consistent with a diagnosis of depression made by independent clinicians [32]. Evidence also shows that adults who were diagnosed with depression based on the PHQ-9 had more impairment, utilised more health care and had more disability days [32]. Each parent completed the PHQ-9 at Times 1 and 2. Mothers’ depressive symptoms correlated with fathers’ depressive symptoms at Times 1 (*r* = .20, *p* = .01) and 2 (*r* = .22, *p* = .03).

#### Parents’ expressed emotion

We used the Pre-school Five Minute Speech Sample [36] to measure expressed emotion in mothers and fathers. A trained researcher asked each parent separately to “talk for five minutes, without any prompts or interruptions from the researcher, about their thoughts and feelings towards their child and their relationship with their child over the last 6 months”. The Pre-school Five Minute Speech Sample (P-FMSS) has three global variables including initial statement (scored as positive, negative and neutral), relationship (scored as positive, neutral and negative), warmth (scored as high, moderate and low) and frequency count of positive and critical comments. Previous research has shown that the scheme has satisfactory reliability and discriminant validity [36]. In this study, we limited the analyses to the frequency count of positive and critical comments because the number of parents who had a negative initial statement, low warmth and negative parent-child relationship was low. The inter-rater reliability was assessed in 40 speech samples and it was high for both critical (ICC = 0.9) and positive comments (ICC = 0.9). All speech samples were blindly coded.

#### Children’s internalizing (emotional) and externalizing (behavioural) problems

Independently, each parent completed the Child Behavior Checklist 1½ to 5 [37] to measure children’s emotional and behavioural problems at Time 1. We then standardised and averaged mothers’ and fathers’ scores on the externalising scale and used this variable for the analyses. Similarly, we averaged mothers’ and fathers’ scores on the internalising scale and used this variable for all subsequent analyses. At Time 2, we used the Child Behavior Checklist 1½ to 5 (CBCL 1½ to 5 years) for those children who were still under 6 years old and the Child Behavior Checklist for ages 6 to 18 years (CBCL 6 to 18 years) for those children aged 6 and above. We used standardised scores to make comparisons between scores independently of which scale parents completed at Time 2. For parents who filled out the externalising scale of the CBCL 1½ to 5 years (*younger version*) at Time 2, we converted each parent’s Time 2 CBCL score into a *z*-score (one within mothers, one within fathers) that we then averaged across parents of each child (there were no significant differences in mothers’ and fathers’ CBCL scores, all *t*s < 1). Separately, for parents who completed the externalising scale of the CBCL 6 to 18 years (*older version*) at Time 2, we converted each parent’s Time 2 CBCL score into a *z*-score (one for mothers, one for fathers) that we then averaged across parents of each child. For each child, the corresponding externalising mean *z*-score at Time 2 was analysed as the outcome variable. The same procedure was used to create the internalizing outcome variable. We used this procedure to remove variability arising from parents completing different versions of the CBCL at Time 2.

#### Covariates/Confounders

Covariates/confounders included children’s gender, emotional and behavioural problems at baseline, and parents’ age and education were measured using parents’ reports. Their selection was based on previous literature showing that children’s male gender, early symptoms of psychopathology and parents’ characteristics (e.g., young age, low level of education) predict adverse child outcomes [4, 6–8].

### Statistical analysis

We used continuous PHQ-9 scores in our main analyses given evidence indicating that the intensity of maternal depressive symptoms (rather than meeting categorical criteria for depression diagnosis) is a sensitive predictor of child outcomes [38]. Mothers’ and fathers’ critical comments at Time 1 and Time 2 were highly positively skewed, so we inverse-transformed these variables at both time points to minimise skew before reflecting the scores to preserve directionality. Analyses were conducted using MPlus version 7. Missing data were imputed using full information maximum likelihood and the MLR estimator was used to provide greater robustness.

First, bivariate correlations were used to examine associations among study variables, followed by linear regressions to test the unique relationship between mothers’ and fathers’ depressive symptoms (predictors) and children emotional and behavioural problems (outcomes). Covariates including child gender (effect coded: –1 = boys, +1 = girls), parent age and education, and baseline child outcomes (emotional and behavioural problems) were included at Step 1. Mothers’ and fathers’ depressive symptoms were entered simultaneously at Step 2. The interaction term (mothers’ x fathers’ depressive symptoms at Time 1) was created by multiplying the centred values for mothers’ and fathers’ depressive symptoms and was entered at Step 3. The interaction terms (mothers’ depressive symptoms at Time 1 x child gender, fathers’ depressive symptoms at Time 1 x child gender) were created by multiplying the centred values for each parent’s depressive symptoms with child’s gender, and entered at Step 4. We also created an interaction term by multiplying together the centred values for mothers’ depressive symptoms, fathers’ depressive symptoms and child’s gender (mothers’ depressive symptoms x fathers’ depressive symptoms at Time 1 x child gender) and entered this at Step 5.

To examine longitudinal associations between parent’s critical / positive comments (expressed emotion constituents) and child’s outcomes (emotional and behavioural problems), we then conducted a series of multivariate regression analyses in which pairs of variables (i.e., one parenting variable and one child outcome variable) at Time 2 were simultaneously regressed on the equivalent variables at Time 1, thereby revealing the cross-lagged associations while controlling for the autoregressive paths (i.e., both variables at Time 1) and confounding variables (child gender, parent age, education, and depressive symptoms). We used an alpha level of .05 for all inferential tests relating to our focal hypotheses. Unpredicted statistically significant results were not interpreted due to the increased risk of Type I error with multiple testing. All relevant data for this paper can be found in the Supporting Information file (S1 File).

## Results

### Descriptive statistics for expressed emotion—Critical and positive comments

Table 1 presents means and standard deviations for (untransformed) expressed emotion critical and positive comments at Times 1 and 2 for mothers and fathers. We conducted paired samples t-tests to examine if there were significant differences in mean levels of positive and critical comments (Times 1 and 2) between mothers and fathers but the results revealed no significant differences: Time 1 positive comments (mother *M* = 8.56, *SD* = 5.17; father *M* = 7.70, *SD* = 5.22), *t*(141) = 1.69, *p* = .09; Time 2 positive comments (mother *M* = 8.01, *SD* = 4.87; father *M* = 7.26, *SD* = 4.28), *t*(92) = 1.32, *p* = .19; Time 1 critical comments (mother *M* = 1.62, *SD* = 2.37; father *M* = 1.45, *SD* = 2.24), *t*(141) = 1.24, *p* = .22; Time 2 critical comments (*M* = 1.74, *SD* = 2.43; father *M* = 1.65, *SD* = 2.03), *t*(92) = 0.20, *p* = .84.

**Table 1:** Means and standard deviations for mothers’ and fathers’ expressed emotion—Critical and positive comments.

	*N*	*M*	*SD*
**Mothers’ critical comments Time 1**	144	1.60	2.36
**Mothers’ positive comments Time 1**	144	8.53	5.14
**Fathers’ critical comments Time 1**	158	1.37	2.16
**Fathers’ positive comments Time 1**	158	7.56	5.10
**Mothers’ critical comments Time 2**	93	1.74	2.43
**Mothers’ positive comments Time 2**	93	8.01	4.87
**Fathers’ critical comments Time 2**	105	1.54	1.98
**Fathers’ positive comments Time 2**	105	7.36	4.34

Descriptive statistics are based on untransformed complete data without imputation.

### Correlations among study variables

Table 2 presents correlations among study variables for mothers. Mothers’ critical comments at Time 1 correlated positively with mothers’ critical comments at Time 2. Similarly, mothers’ positive comments at Time 1 correlated positively with positive comments at Time 2. Mothers’ depressive symptoms at Time 1 correlated positively with child emotional and behavioural problems (Time 1), behavioural problems (Time 2) and mothers’ depressive symptoms at Time 2. Similarly, mothers’ depressive symptoms at Time 2 correlated positively with child emotional and behavioural problems (Times 1 and 2). In addition, mothers’ depressive symptoms (Time 2) correlated negatively with positive comments (Time 2). Apart from the non-significant correlations between mothers’ critical comments (Time 1) and children’s emotional problems (Time 1) and between mothers’ positive comments (Time 1) and children’s behavioural problems (Time 2), all other correlations between mothers’ expressed emotion constituents and children’s outcomes were significant in the expected direction.

**Table 2:** Correlations among study variables for mothers.

	**1**	**2**	**3**	**4**	**5**	**6**	**7**	**8**	**9**	**10**
**1 Mothers’ depressive symptoms Time 1**										
**2 Mothers’ depressive symptoms Time 2**	.59***									
**3 Children’s emotional problems Time 1**	.24*	.38***								
**4 Children’s behavioural problems Time 1**	.24***	.34***	.48***							
**5 Children’s emotional problems Time 2**	.25	.30***	.66***	.34***						
**6 Children’s behavioural problems Time 2**	.30*	.29*	.40***	.70***	.54***					
**7 Mothers’ critical comments Time 1**	.12	.17	.15	.47***	.28***	.44***				
**8 Mothers’ positive comments Time 1**	-.05	-.12	-.26***	-.22**	-.24***	-.20	-.30***			
**9 Mothers’ critical comments Time 2**	.13	.16	.28**	.43***	.41***	.64***	.40***	-.27**		
**10 Mothers’ positive comments Time 2**	-.13	-.24**	-.51***	-.30***	-.47***	-.39***	-.21*	.53***	-.37***	

Sample sizes vary due to missing data.

*** *p* < .001,

** *p* < .01;

* *p* < .05.

Table 3 presents correlations among study variables for fathers. Fathers’ positive (but not critical) comments were significantly correlated between Times 1 and 2. Fathers’ depressive symptoms at Time 1 correlated positively with child emotional and behavioural problems at Time 2 and fathers’ depressive symptoms at Time 2. Similarly, fathers’ depressive symptoms at Time 2 correlated positively with child emotional problems (Times 1 and 2). Fathers’ positive and critical comments correlated with child outcomes in the expected direction apart from the non-significant correlations between fathers’ critical comments (Time 1 and 2) and child emotional problems (Time 1), between child emotional problems (Time 1) and fathers’ positive comments (Time 2), and between fathers’ critical comments (Time 1) and child emotional problems (Time 2).

**Table 3:** Correlations among study variables for fathers.

	**1**	**2**	**3**	**4**	**5**	**6**	**7**	**8**	**9**	**10**
**1 Fathers’ depressive symptoms Time 1**										
**2 Fathers’ depressive symptoms Time 2**	.55***									
**3 Children’s emotional problems Time 1**	.13	.26*								
**4 Children’s behavioural problems Time 1**	.15	.06	.48***							
**5 Children’s emotional problems Time 2**	.25*	.28*	.66***	.34***						
**6 Children’s behavioural problems Time 2**	.23*	.28	.40***	.70***	.54***					
**7 Fathers’ critical comments Time 1**	.08	-.09	.07	.36***	.10	.35***				
**8 Fathers’ positive comments Time 1**	.04	-.03	-.17*	-.25***	-.34***	-.36***	-.25***			
**9 Fathers’ critical comments Time 2**	.01	-.01	.20	.36***	.36***	.53***	.06	-.04		
**10 Fathers’ positive comments Time 2**	-.07	-.02	-.17	-.26**	-.35***	-.37***	-.34***	.38***	-.24*	

Sample sizes vary due to missing data.

*** *p* < .001;

** *p* < .01;

* *p* < .05.

### Concurrent associations between parents’ depressive symptoms and children’s outcomes

Next, we ran multiple regression analyses to examine the unique and interactive associations between parents’ depressive symptoms and child emotional and behavioural problems at Time 1 after controlling for confounding variables. In these models, we also examined the interactive associations between mothers’ x fathers’ depressive symptoms, mothers’/father’s depressive symptoms x child gender and the three-way interaction between mothers’ depressive symptoms, fathers’ depressive symptoms and child gender on child outcomes at Time 1. Parents’ age and education were included as covariates in Step 1 and mothers’ and fathers’ depressive symptoms were entered simultaneously in Step 2. The interaction between mothers’ and fathers’ depressive symptoms was entered in Step 3. The interactions between mothers’ depressive symptoms and child gender, and between fathers’ depressive symptoms and child gender were entered in Step 4. Finally, the three-way interaction between mothers’ depressive symptoms, fathers’ depressive symptoms and child gender was entered in Step 5. Table 4 shows the results of all regression models.

**Table 4:** Concurrent associations between parents’ depressive symptoms and child outcomes (emotional and behavioural problems) at Time 1.

			Child emotional problems Time 1				Child behavioural problems Time 1	
	*N*	*R*^*2*^	*β*	*p*	*N*	*R*^*2*^	*β*	*p*
***Step 1***	138	.06			138	.03		
**Child’s gender (-1 = Male, 1 = Female)**			.17	.04			.07	.48
**Mothers’ age**			-.17	.11			-.17	.15
**Fathers’ age**			.06	.56			.09	.39
**Mothers’ education (-1 = No Degree, 1 = Degree)**			-.08	.46			-.05	.61
**Fathers’ education (-1 = No Degree, 1 = Degree)**			-.02	.89			-.02	.84
***Step 2***	138	.10			138	.08*		
**Child’s gender (-1 = Male, 1 = Female)**			.15	.05			.05	.60
**Mothers’ age**			-.14	.16			-.15	.22
**Fathers’ age**			.04	.70			.07	.53
**Mothers’ education (-1 = No Degree, 1 = Degree)**			-.06	.60			-.03	.79
**Fathers’ education (-1 = No Degree, 1 = Degree)**			-.03	.82			-.03	.77
**Mothers’ depressive symptoms Time 1**			.19	.07			.21	.007
**Fathers’ depressive symptoms Time 1**			.07	.41			.08	.29
***Step 3***	135	.11*			135	.08*		
**Child’s gender (-1 = Male, 1 = Female)**			.16	.05			.05	.61
**Mothers’ age**			-.13	.22			-.14	.24
**Fathers’ age**			.04	.69			.07	.52
**Mothers’ education (-1 = No Degree, 1 = Degree)**			-.06	.58			-.03	.81
**Fathers’ education (-1 = No Degree, 1 = Degree)**			-.01	.95			-.03	.82
**Mothers’ depressive symptoms Time 1**			.16	.051			.20	.01
**Fathers’ depressive symptoms Time 1**			.02	.81			.07	.38
**Mothers X Fathers depressive symptoms at Time 1**			.19	.03			.05	.46
***Step 4***	135	.15*			135	.09*		
**Child’s gender (-1 = Male, 1 = Female)**			.17	.02			.06	.53
**Mothers’ age**			-.14	.17			-.15	.21
**Fathers’ age**			.07	.54			.09	.44
**Mothers’ education (-1 = No Degree, 1 = Degree)**			-.07	.56			-.03	.80
**Fathers’ education (-1 = No Degree, 1 = Degree)**			.01	.96			-.02	.88
**Mothers’ depressive symptoms Time 1**			.12	.14			.19	.02
**Fathers’ depressive symptoms Time 1**			-.01	.88			.04	.60
**Mothers X Fathers depressive symptoms at Time 1**			.16	.02			.03	.64
**Mothers depressive symptoms X Child gender**			.13	.050			.04	.67
**Fathers depressive symptoms X Child gender**			.14	.04			.11	.16
***Step 5***	135	.15*			135	.09*		
**Child’s gender (-1 = Male, 1 = Female)**			.16	.04			.06	.53
**Mothers’ age**			-.14	.16			-.15	.21
**Fathers’ age**			.07	.54			.09	.44
**Mothers’ education (-1 = No Degree, 1 = Degree)**			-.07	.54			-.03	.80
**Fathers’ education (-1 = No Degree, 1 = Degree)**			.001	.99			-.02	.88
**Mothers’ depressive symptoms Time 1**			.12	.16			.19	.01
**Fathers’ depressive symptoms Time 1**			-.01	.93			.04	.61
**Mothers X Fathers depressive symptoms at Time 1**			.12	.17			.04	.73
**Mothers depressive symptoms X Child gender**			.13	.08			.04	.67
**Fathers depressive symptoms X Child gender**			.13	.08			.11	.18
**Mothers X fathers depressive symptoms X Child gender**			.06	.42			-.01	.97

Due to missing data that could not be imputed in later steps of the regression model, sample size was reduced in Steps 3–5.

* *p* < .05 for model fit. The use of the MLR algorithm means that it was not possible to test the significance of improvement in model fit.

To check the clinical validity of the results using the PHQ-9, we repeated this analyses using a categorical variable of depression (-1 = nondepressed; 1 = depressed) assessed with the Structured Clinical Interview for DSM-IV [34]. When parents’ depressive symptoms were replaced with this research diagnosis of depression, the results revealed significant interactions between mothers’ depression and children’s gender in predicting children’s emotional problems (β = .18, *p* < .05) and behavioural problems (β = .18, *p* < .05). There was also a significant interaction between fathers’ depression and children’s gender in predicting children’s emotional problems (β = .18, *p* < .05).

In Step 1, only child gender explained unique variance in child emotional problems at Time 1, with girls scoring higher than boys. In Step 2, neither mothers’ nor fathers’ depressive symptoms at Time 1 significantly predicted child emotional problems at Time 1. However, Step 3 revealed a significant interaction between mothers’ and fathers’ depressive symptoms at Time 1. This interaction is plotted in S1 Fig for values of mothers’ and fathers’ depressive symptoms that are one standard deviation above and below the mean and indicates that fathers’ depressive symptoms were associated with more child emotional problems when mothers were high in depressive symptoms, simple slope = .17, *t*(125) = 1.98, *p* < .05. Fathers’ depressive symptoms were not associated with child emotional problems for mothers who were low in depressive symptoms, simple slope = -.13, *t*(125) = 1.08, *p* = .28).

In Step 4, mothers’ depressive symptoms at Time 1 did not significantly interact with child gender in predicting child emotional problems but fathers’ depressive symptoms did significantly interact with child gender in predicting child emotional problems. This interaction is plotted in S2 Fig for boys and girls at values of fathers’ depressive symptoms that are one standard deviation above and below the mean, and indicates that the concurrent relationship between fathers’ depressive symptoms and child emotional problems differed between boys and girls (S2 Fig). However, the relationship between fathers’ depressive symptoms and child emotional problems at Time 1 was not significant for either boys (simple slope = -.17, *t*(123) = -1.44, *p* = .15) or girls (simple slope = .15, *t*(123) = 1.34, *p* = .18).

Step 5 revealed that there was no significant three-way interaction between mothers’ depressive symptoms, fathers’ depressive symptoms and child gender.

We repeated this hierarchical regression analysis with child behavioural problems as the outcome. As shown in Table 4, parental age and education did not predict unique variance in child behavioural problems at Time 1. In Step 2, mothers’ (but not fathers’) depressive symptoms at Time 1 were significantly positively associated with child behavioural problems at Time 1. However, there was no significant interaction between mothers’ and fathers’ depressive symptoms in Step 3, and no significant interactions between mothers’ and fathers’ depressive symptoms and child gender in Steps 4 and 5.

### Longitudinal associations between parents’ depressive symptoms and children’s outcomes

Next we conducted hierarchical regression analysis to examine whether mothers’ and fathers’ depressive symptoms at Time 1 would each uniquely and interactively predict child emotional and behavioural problems at Time 2. Child gender, child outcome at Time 1 and parents’ age and education were entered at Step 1, and mothers’ and fathers’ depressive symptoms were entered at Step 2. The interaction between mothers’ and fathers’ depressive symptoms was entered at Step 3, followed by the interactions between mothers’ depressive symptoms and child gender, and between fathers’ depressive symptoms and child gender at Step 4. The three-way interaction term between mothers’ depressive symptoms, fathers’ depressive symptoms and child gender was entered at step 5. Results are presented in Table 5.

**Table 5:** Longitudinal associations between parents’ depressive symptoms and child outcomes (emotional and behavioural problems) at Time 2.

			Child emotional problems Time 2				Child behavioural problems Time 2	
	N	*R*^*2*^	*β*	*p*	N	*R*^*2*^	*β*	*p*
***Step 1***	138	.50***			138	.50***		
**Children’s outcome at Time 1**			.68	< .001			.70	< .001
**Children’s gender (-1 = Male, 1 = Female)**			.04	.62			.12	.12
**Mothers’ age**			.28	.004			.02	.85
**Fathers’ age**			-.15	.09			.02	.86
**Mothers’ education (-1 = No Degree, 1 = Degree)**			-.15	.12			-.09	.34
**Fathers’ education (-1 = No Degree, 1 = Degree)**			.04	.67			.11	.30
***Step 2***	138	.56***			138	.55***		
**Children’s outcome at Time 1**			.62	< .001			.66	< .001
**Children’s gender (-1 = Male; 1 = Female)**			.03	.61			.11	.12
**Mothers’ age**			.28	.004			.02	.76
**Fathers’ age**			-.23	.02			-.05	.60
**Mothers’ education (-1 = No Degree, 1 = Degree)**			-.12	.22			-.07	.48
**Fathers’ education (-1 = No Degree, 1 = Degree)**			.02	.82			.10	.36
**Mothers’ depressive symptoms Time 1**			.14	.17			.09	.33
**Fathers’ depressive symptoms Time 1**			.19	.01			.17	.02
***Step 3***	135	.56***			135	.55***		
**Children’s outcome at Time 1**			.61	< .001			.65	< .001
**Children’s gender (-1 = Male, 1 = Female)**			.04	.55			.11	.12
**Mothers’ age**			.28	.003			.03	.72
**Fathers’ age**			-.25	.01			-.06	.56
**Mothers’ education (-1 = No Degree, 1 = Degree)**			-.13	.18			-.07	.46
**Fathers’ education (-1 = No Degree, 1 = Degree)**			.01	.88			.09	.38
**Mothers’ depressive symptoms Time 1**			.15	.11			.10	.33
**Fathers’ depressive symptoms Time 1**			.18	.02			.17	.02
**Mothers X Fathers depressive symptoms at Time 1**			.08	.56			.06	.79
***Step 4***	135	.56***			135	.55***		
**Children’s outcome at Time 1**			.60	< .001			.63	< .001
**Children’s gender (-1 = Male, 1 = Female)**			.05	.46			.13	.10
**Mothers’ age**			.28	.003			.02	.77
**Fathers’ age**			-.24	.01			-.04	.70
**Mothers’ education (-1 = No Degree, 1 = Degree)**			-.14	.17			-.08	.41
**Fathers’ education (-1 = No Degree, 1 = Degree)**			.01	.95			.08	.43
**Mothers’ depressive symptoms Time 1**			.14	.14			.08	.50
**Fathers’ depressive symptoms Time 1**			.16	.05			.14	.13
**Mothers X Fathers depressive symptoms at Time 1**			.12	.38			.13	.58
**Mothers depressive symptoms X Child gender**			.06	.55			.10	.46
**Fathers depressive symptoms X Child gender**			.09	.23			.12	.19
***Step 5***	135	.64***			135	.64***		
**Children’s outcome at Time 1**			.54	< .001			.57	< .001
**Children’s gender (-1 = Male, 1 = Female)**			.02	.75			.09	.23
**Mothers’ age**			.24	.007			.01	.94
**Fathers’ age**			-.22	.02			-.03	.75
**Mothers’ education (-1 = No Degree, 1 = Degree)**			-.16	.08			-.11	.21
**Fathers’ education (-1 = No Degree, 1 = Degree)**			.02	.84			.09	.36
**Mothers’ depressive symptoms Time 1**			.21	.008			.15	.14
**Fathers’ depressive symptoms Time 1**			.15	.03			.13	.09
**Mothers X Fathers depressive symptoms at Time 1**			.05	.56			.06	.71
**Mothers depressive symptoms X Child gender**			.04	.66			.07	.50
**Fathers depressive symptoms X Child gender**			.08	.19			.11	.14
**Mothers X fathers depressive symptoms X Child gender**			.30	.02			.31	.06

Due to missing data that could not be imputed in later steps of the regression model, sample size was reduced in Steps 3–5.

*** *p* < .001 for model fit. The use of the MLR algorithm means that it was not possible to test the significance of improvement in model fit.

When parents’ depressive symptoms were replaced with a research diagnosis of depression, the results revealed that mothers’ depression at Time 1 predicted child emotional problems (β = .23, *p* = .006).

In the model predicting child emotional problems at Time 2, child emotional problems at Time 1 and greater mother age predicted greater child emotional problems at Time 2. When entered in Step 2, fathers’ (but not mothers’) depressive symptoms at Time 1 were uniquely and significantly associated with greater child emotional problems at Time 2. Step 3 revealed no significant interaction between mothers’ and fathers’ depressive symptoms at Time 1 in predicting child emotional problems at Time 2. In Step 4, mothers’ and fathers’ depressive symptoms at Time 1 did not interact with child gender in predicting child emotional problems at Time 2, but the three-way interaction between mothers’ depressive symptoms, fathers’ depressive symptoms and child gender was a significant predictor in Step 5. S3 and S4 Figs plot this interaction for boys and girls at values of mothers’ and fathers’ depressive symptoms that are one standard deviation above and below the mean. S3 Fig shows that the association between fathers’ depressive symptoms at Time 1 and changes in boys’ emotional problems at Time 2 was positive (simple slope = .49, *t*(121) = 16.56, *p* < .001) when mothers’ depressive symptoms at Time 1 were low and negative (slope = -.21, *t*(121) = 8.09, *p* < .001) when mothers’ depressive symptoms at Time 1 were high. At average levels of mothers’ depressive symptoms, the association between fathers’ depressive symptoms and change in boys’ emotional problems was significant (simple slope = .14, *t*(121) = 4.73, *p* < .001).

S4 Fig shows that the association between fathers’ depressive symptoms at Time 1 and changes in girls’ emotional problems at Time 2 was significant and positive when mothers were high on depressive symptoms at Time 1 (simple slope = .94, *t*(121) = 37.64, *p* < .001) but was not significant when mothers were low on depressive symptoms (simple slope = -.01, *t*(121) = -.34, *p* = .73) (S4 Fig). At average levels of mothers’ depressive symptoms, the slope between fathers’ depressive symptoms at Time and change in girls’ emotional problems was positive and significant (slope = .47, *t*(121) = 18.62, *p* < .001). Thus, although fathers’ depressive symptoms at Time 1 were associated with deterioration in emotional problems at Time 2, this effect was most pronounced for girls whose mothers were also high in depressive symptoms at Time 1.

We repeated this hierarchical regression analysis to predict child behavioural problems at Time 2. In Step 1, child behavioural problems at Time 1 explained unique variance in child behavioural problems at Time 2, but parental age and education did not. In Step 2, fathers’ (but not mothers’) depressive symptoms at Time 1 were significantly and positively associated with child behavioural problems at Time 2. Step 3 revealed that mothers’ and fathers’ depressive symptoms at Time 1 did not interact to predict child behavioural problems at Time 2. In Steps 4 and 5, mothers’ and fathers’ depressive symptoms in Time 1 did not interact significantly with child gender to predict child behavioural problems at Time 2.

### Associations between parents’ expressed emotion constituents (positive and critical comments) and children’s emotional and behavioural problems

We next examined longitudinal multivariate (path) models investigating autoregressive and cross-lagged associations between parental comments and child problems over time. Table 6 shows that autoregressive paths for positive parental comments were significant in all models, although, critical comments only showed significant autoregressive relationships in the model involving mothers and child emotional problems. Significant autoregressive paths were found for child emotional and behavioural problems in all models.

**Table 6:** Cross-lagged and autoregressive associations between parents’ positive and critical comments and children’s outcomes.

Mothers	*N*	*β*	*p*	Fathers	*N*	*β*	*p*
**T1 M_CC = > T2 EXT**	141	.01	.94	**T1 F_CC = > T2 EXT**	157	.02	.83
**T1 EXT = > T2 M_CC**		.32	.002	**T1 EXT = > T2 F_CC**		.46	< .001
**M_CC autoregressive**		.18	.11	**F_CC autoregressive**		-.10	.30
**EXT autoregressive**		.66	< .001	**EXT autoregressive**		.67	< .001
**T1 M_CC = > T2 INT**	141	.08	.27	**T1 F_CC = > T2 INT**	157	-.08	.37
**T1 INT = > T2 M_CC**		.15	.15	**T1 INT = > T2 F_CC**		.23	.02
**M_CC autoregressive**		.33	.001	**F_CC autoregressive**		.05	.64
**INT autoregressive**		.64	< .001	**INT autoregressive**		.63	< .001
**T1 M_PC = > T2 EXT**	141	.03	.66	**T1 F_PC = > T2 EXT**	157	-.17	.002
**T1 EXT = > T2 M_PC**		-.13	.18	**T1 EXT = > T2 F_PC**		-.18	.07
**M_PC autoregressive**		.51	< .001	**F_PC autoregressive**		.32	< .001
**EXT autoregressive**		.67	< .001	**EXT autoregressive**		.65	< .001
**T1 M_PC = > T2 INT**	141	-.04	.44	**T1 F_PC = > T2 INT**	157	-.20	.001
**T1 INT = > T2 M_PC**		-.40	< .001	**T1 INT = > T2 F_PC**		-.11	.21
**M_PC autoregressive**		.42	< .001	**F_PC autoregressive**		.35	< .001
**INT autoregressive**		.65	< .001	**INT autoregressive**		.58	< .001

In all of these models, we also included child gender, and depressive symptoms, educational attainment and age of the relevant parent as covariates (for presentational clarity, we do not include these coefficients here). The pattern of significant findings was identical when a categorical research diagnosis of depression was used instead of the continuous measure of depression symptoms (PHQ-9 score).

INT = internalizing (emotional problems), EXT = Externalizing (behavioural problems); M = Mothers, F = Fathers; CC = Critical comments, PC = Positive comments; T1 = Time 1, T2 = Time 2; autoregressive = T1 variable predicting T2 variable.

For mothers, positive and critical comments at Time 1 did not predict change in child emotional and behavioural problems. Child behavioural problems at Time 1 predicted change in mothers’ critical comments but did not significantly predict change in mothers’ positive comments. Child emotional problems at Time 1 predicted change in positive comments over time, but did not predict change in critical comments. For fathers, critical comments at Time 1 did not significantly predict change in child emotional and behavioural problems but child emotional and behavioural problems at Time 1 predicted change in fathers’ critical comments at Time 2. Child emotional problems at Time 1 did not predict change in fathers’ positive comments but the association between child behavioural problems and change in fathers’ positive comments only approached significance. Fathers’ positive comments at Time 1 predicted change in emotional and behavioural problems at Time 2.

## Discussion

The findings provide partial support for the study’s hypotheses. Fathers’ depressive symptoms predicted children’s emotional and behavioural problems later in life even when accounting for children’s problems at baseline, maternal depressive symptoms, children’s gender, and parents’ education and age. For mothers, depressive symptoms were concurrently associated with behavioural problems among their children but did not predict such problems longitudinally. Importantly, fathers’ depressive symptoms were concurrently associated with more emotional problems among their children only when mothers had high levels of depressive symptoms. Our study did not provide support for the hypothesis that the concurrent association between parental depressive symptoms and child outcomes is stronger for same-gendered children. Longitudinally, however, the relationship between fathers’ depressive symptoms at baseline and later child outcomes was moderated by gender and mothers’ depressive symptoms. For girls, fathers’ greater depressive symptoms were associated with greater deterioration in emotional problems when mothers were higher in depressive symptoms. These findings are consistent with a limited number of studies showing that children of two depressed parents have worse outcomes compared to children with one or no depressed parent [13, 38]. For boys, fathers’ depressive symptoms at baseline were associated with greater deterioration in emotional problems when mothers scored low on depressive symptoms while fathers’ depressive symptoms were associated with less deterioration in emotional problems in boys when their mothers scored high on depressive symptoms. This finding was unexpected and difficult to interpret using previous theory. However, these interactions should be interpreted with caution given the limited sample size of this study. Taken together, our findings underscore the importance of adopting a family unit approach when examining child mental health and also considering child gender as a potential moderator of the link between parent’s depression and adverse child’s outcomes. With regard to causal processes, theoretical models and empirical studies have stressed both genetic and environmental mechanisms [14–15] that may explain the link between parental depression and child psychopathology. Recent work has shown that fathers’ depressive symptoms at 9 months predicted emotion regulation problems in their children at 7 years and this association was explained by a conflicted father-child relationship during the preschool years [39]. Studies also indicate that the link between parental depression and child psychopathology might be explained by different environmental mechanisms in depressed mothers and fathers [40]. These mechanisms need to be tested in future research.

The study provided limited support for bi-directional links between fathers’ positive comments and children’s behavioural problems. Fathers’ positive comments at Time 1 predicted change in child behavioural problems at Time 2 but the reverse path from child behavioural problems at Time 1 to fathers’ positive comments at Time 2 only approached significance. In other words, there was some evidence suggesting that fathers’ positive comments and children’s behavioural problems predict each other over time. Such a feedback cycle would support the transactional model [23]. However, these findings should be interpreted with caution because the path from Time 1 child behavioural problems to Time 2 fathers’ positive comments was only a trend. It is also worth noting that this bi-directional association was based on parents’ reports on their children’s outcomes rather than children’s actual problems. It is possible that parents’ perceptions of their children’s earlier problems may have influenced their mood and the number of comments they make about their child. Our study did not provide support for bi-directional relationships for mothers, with no evidence emerging for associations between child outcomes at baseline and parental comments at follow-up. It is possible that mothers and fathers have different effects on their child and might be influenced differently by their child’s behaviour. Whether fathers’ and mothers’ expressed emotion and depression have distinct influences on child mental health is an important topic for future research. There were no significant bi-directional associations between parents’ critical/positive comments and children’s emotional problems. This may be because parents’ responses to their children’s emotional difficulties may be less intense than for behavioural difficulties such that bi-directional associations are less likely to occur. The study’s findings revealed that the child’s behaviour was associated with change in mothers’ and fathers’ expressed emotion constituents and provided support for child-driven effects. In other words, although we assumed parent-to-child effects, difficult children’s behaviour may also influence parents’ behaviour (i.e., reverse causality). In addition, fathers’ positive comments predicted decreased child emotional and behavioural problems over time. Children whose fathers often make positive comments may be raised in more functional family environments and have better relationship with their fathers. Other studies have also provided evidence consistent with the positive influences of fathers in their children’s lives [41–43].

Finally, correlational analyses provided support for a negative cross-sectional association between mothers’ depressive symptoms and mothers’ positive comments at Time 2 but not at Time 1. Previous studies have found links between maternal depression and higher expressed emotion [30–31] but further research is needed to establish if there is a consistent association. Positive comments at Time 1 were also significantly associated with positive comments at Time 2 for both mothers and fathers while correlations for critical comments between Times 1 and 2 were only significant for mothers. The current literature is inconsistent about the stability of expressed emotion over time. In a longitudinal study, Richards *et al*. [44], found no support for the stability of expressed emotion. The authors described expressed emotion as a “momentary state measure” (p. 9) that tends to fluctuate according to children’s psychopathology and development. Further research is needed to test the stability of expressed emotion over time.

The study had a number of limitations. The sample consisted of relatively well-educated parents and thus the results should be generalised to other populations cautiously. Parents self-reported on child outcomes and depressive symptoms using questionnaires, such that their reports may be subject to shared method variance. Parents with depression could have reported higher levels of child emotional and behavioural problems [45–46]. Future studies should include independent reports of child emotional and behavioural problems. While the results are statistically significant, the coefficients of determination (*R*-squared) indicating how well these data fit the models are small, suggesting that parental depression is one out of several factors (environmental and genetic) that predict increased problems in their children. In this study, the effect sizes of maternal and paternal depression on child problems were also small in Cohen’s [47] terms but comparable to effects found elsewhere [48]. Finally, parents’ expressed emotion (positive and critical comments) was expressed in the researcher’s presence and therefore may not have reflected their relationships with their children in the privacy of their own homes. However, empirical evidence indicates that parents scoring high on the critical dimension of expressed emotion displayed more negativity, harshness and unresponsiveness in interactions with their children [49]. Despite the limitations, the study has notable strengths. It assessed the study variables longitudinally, included both mothers and fathers, and speech samples used to measure expressed emotion were coded blind to parents’ characteristics.

### Conclusion

Fathers’ depressive symptoms were concurrently associated with emotional problems in their children only when mothers had high levels of depressive symptoms. Fathers’ depressive symptoms also longitudinally predicted increased emotional and behavioural problems in their children when controlling for maternal depressive symptoms and other important confounders. There was limited evidence for bi-directional relationships between fathers’ positive comments and children’s behavioural problems but no other bi-directional associations were significant for mothers and fathers. If the findings are replicated in future research, they have considerable theoretical and clinical implications. Theoretically, the findings emphasise the importance of considering both parents’ mental health when examining children’s outcomes, and provide limited support for transactional models in which parents and children influence each other’s behaviours. Clinically, the findings stress the importance of screening and treating depression in both parents rather than focusing on mothers only if, of course, there are available services. These interventions and services could become more effective if targeted at both mothers and fathers [50].

## Supporting information

S1 Fig Concurrent relationship between fathers’ depressive symptoms, mothers’ depressive symptoms and children’s emotional problems (internalizing symptoms) at Time 1. (PDF)

S2 Fig Concurrent relationship between fathers’ depressive symptoms and emotional problems (internalizing symptoms) in boys and girls at Time 1. (PDF)

S3 Fig Longitudinal associations between fathers’ depressive symptoms, mothers’ depressive symptoms and emotional problems (internalizing symptoms) in boys at Time 2. (PDF)

S4 Fig Longitudinal associations between fathers’ depressive symptoms, mothers’ depressive symptoms and emotional problems (internalizing symptoms) in girls at Time 2. (PDF)

S1 File Spreadsheet for study variables. (XLSX)
