# Supplementary figures and images for: Parental depressive symptoms, children’s emotional and behavioural problems, and parents’ expressed emotion—Critical and positive comments
Source: PLoS One. 2017 Oct 18;12(10):e0183546. doi: 10.1371/journal.pone.0183546 (PMC5646775; doi:10.1371/journal.pone.0183546)

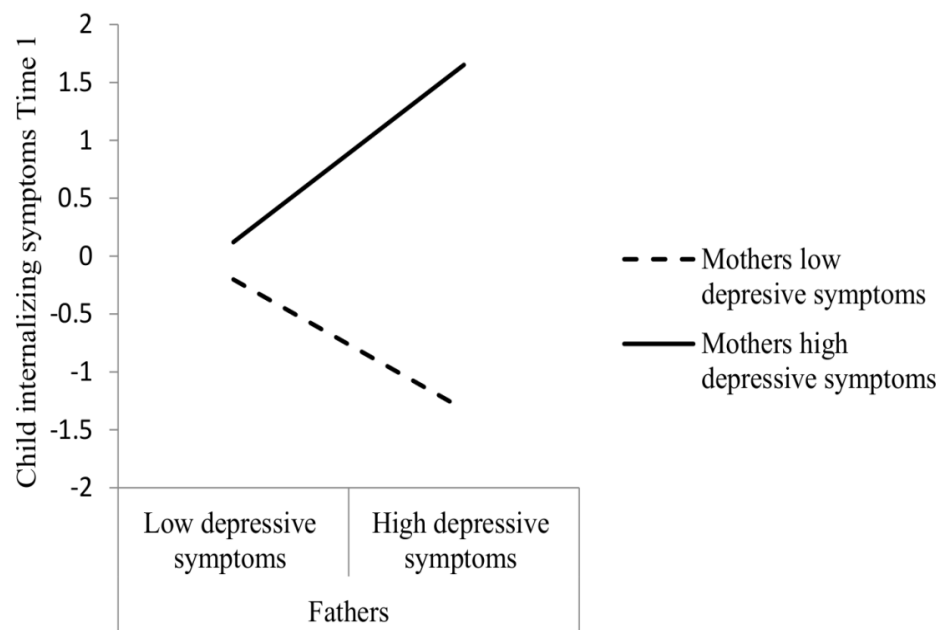

Supplement: S1 Fig — (PDF) [file pone.0183546.s001.pdf]

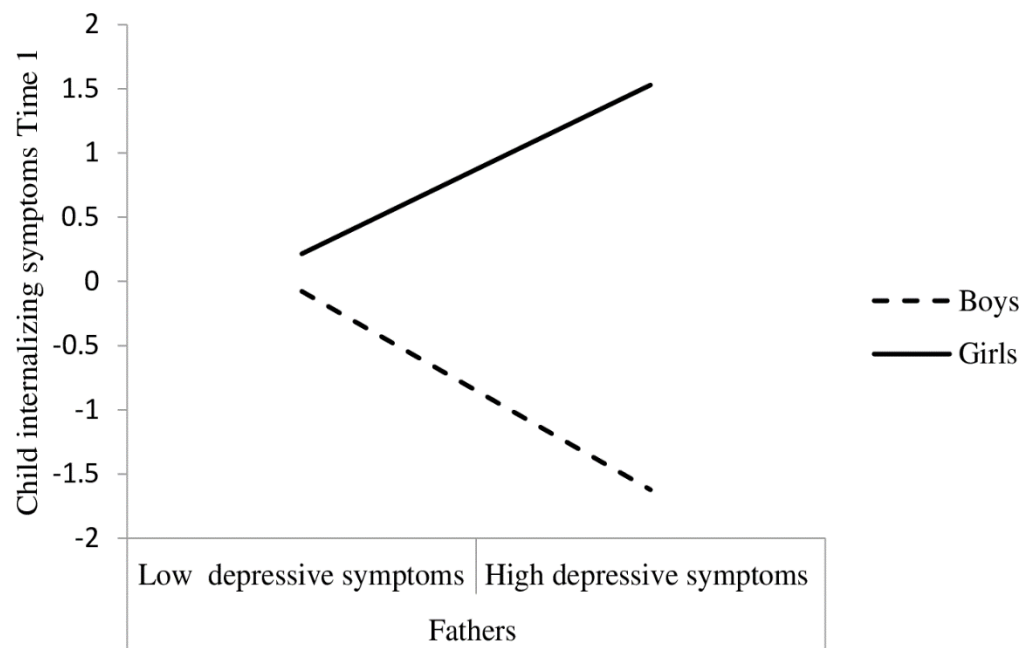

Supplement: S2 Fig — (PDF) [file pone.0183546.s002.pdf]

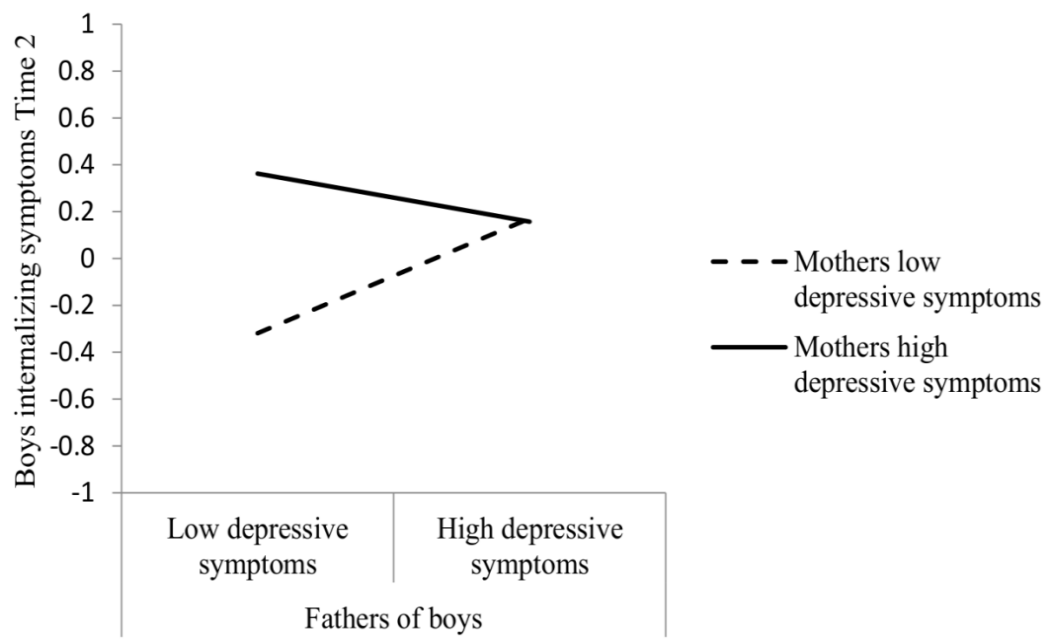

Supplement: S3 Fig — (PDF) [file pone.0183546.s003.pdf]

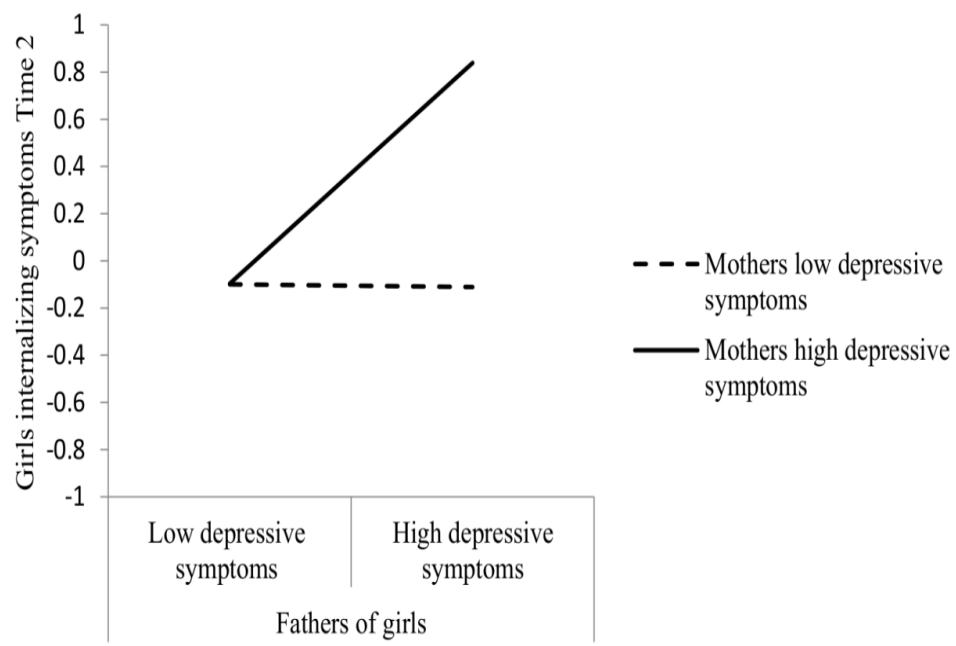

Supplement: S4 Fig — (PDF) [file pone.0183546.s004.pdf]
